# Volatility in the effective size of a freshwater gastropod population

**DOI:** 10.1002/ece3.3912

**Published:** 2018-02-07

**Authors:** Robert T. Dillon

**Affiliations:** ^1^ Department of Biology College of Charleston Charleston SC USA

**Keywords:** allozyme frequencies, effective population size, *Physa acuta*, population subdivision, pulmonate snails, simultaneous hermaphrodite

## Abstract

Despite the utility of gastropod models for the study of evolutionary processes of great generality and importance, their effective population size has rarely been estimated in the field. Here, we report allele frequency variance at three allozyme‐encoding loci monitored over 7 years in a population of the invasive freshwater pulmonate snail *Physa acuta* (Draparnaud 1805), estimating effective population size with both single‐sample and two‐sample approaches. Estimated *N*_e_ declined from effectively infinite in 2009 to approximately 40–50 in 2012 and then rose back to infinity in 2015, corresponding to a striking fluctuation in the apparent census size of the population. Such volatility in *N*_e_ may reflect cryptic population subdivision.

## 1 INTRODUCTION

Since the introduction of the concept by Wright (1931), effective population size (*N*_e_) has been adopted as a parameter in scores of evolutionary models, adaptive and neutral alike (Crow, 2010). The concept has found important applications in animal breeding (Caballero, Santiago, & Toro, 1996) and in conservation biology (Nunney & Elam, 1994). Two categories of methods to estimate *N*_e_ from field data were developed in the early 1980s, a single‐sample approach based on disequilibrium between alleles at unlinked loci and a two‐sample approach based on variance in allelic frequencies between generations (Caballero, 1994). Several single‐sample methods not relying on linkage disequilibrium have more recently been proposed.

Despite the importance of the concept, however, effective population size has rarely been estimated for any gastropod population in the field. Crow and Morton (1955) used variance in progeny number to estimate the *N*_e_/*n* ratio in a laboratory culture of the freshwater pulmonate *Lymnaea (Pseudosuccinea) columella*. The earliest field estimates were those of Murray (1964) and Greenwood (1974), who applied simple single‐sample approaches to shell color polymorphism in an English population of the important land snail model *Cepaea nemoralis*. But another thirty years would elapse before estimates of effective population size were offered for other land snail populations, those of Arnaud and Laval (2004) using microsatellite markers and a two‐sample method, and Ursenbacher, Alvarez, Armbruster, and Baur (2010) using a one‐sample approach.

We are aware of two estimates of the effective size of marine gastropod populations, both inhabiting the European intertidal. Fernandez et al. (2005) followed variation at allozyme‐encoding loci over 14 years in incipient species of *Littorina saxatilis*, estimating effective population size using a two‐sample approach. Riquet, Le Cam, Fonteneau, and Viard (2016) analyzed microsatellite variation in an invasive population of *Crepidula fornicata* over 9 years, comparing both one‐sample and two‐sample estimates.

In the freshwater gastropods, Meunier, Hurtrez‐Bousses, Durand, Rondelaud, and Renaud (2004) used both one‐sample and two‐sample analyses of microsatellite polymorphism to estimate the effective sizes of six French populations of the (predominantly self‐fertilizing) pulmonate *Lymnaea (Galba) truncatula*. Microsatellites and two‐sample techniques were also used by Trouve, Degen, and Goudet (2005) on six populations of *L. truncatula* sampled from Switzerland. The literature contains two single‐sample microsatellite studies on Chinese populations of viviparid snails—*Bellamya quadrata* (Gu, Zhang, et al., 2015) and *B. purificata* (Gu, Zhou, et al., 2015).

**Figure 1:**
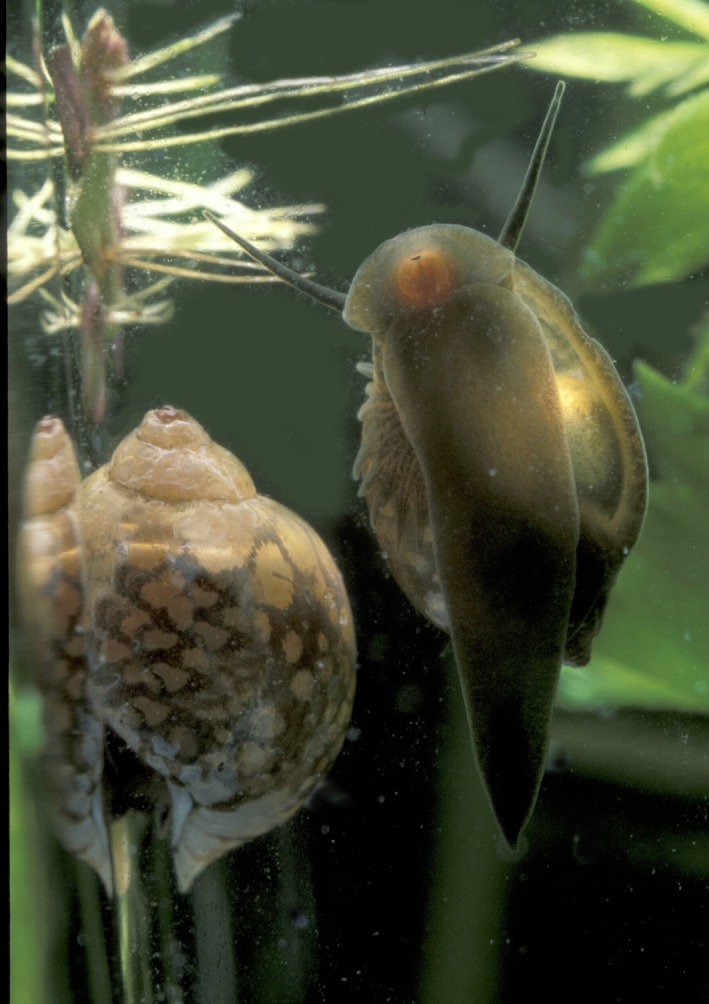
*Physa acuta* (9 mm shell length), courtesy D. Liebman

In recent years, the freshwater basommatophoran pulmonate snail *Physa acuta* has found widespread use as a model organism for a variety of evolutionary studies (Figure 1). Populations of *P. acuta* in both field and laboratory settings have played important roles in studies of mating behavior (Janicke, Vellnow, Lamy, Chapuis, & David, 2014; Janicke, Vellnow, Sarda, & David, 2013; Wethington & Dillon, 1996), sex allocation (Janicke & Chapuis, 2016; Wethington & Dillon, 1993), inbreeding depression (Jarne, Perdieu, Pernot, Delay, & David, 2000; Noel et al., 2016), reproductive isolation (Dillon, Robinson, & Wethington, 2007), gene flow (Bousset, Henry, Sourrouille, & Jarne, 2004; Van Leeuwen et al., 2013), speciation (Dillon, Wethington, & Lydeard, 2011), and ecophenotypic plasticity (Auld & Relyea, 2011; Dillon & Jacquemin, 2015; Gustafson, Kensinger, Bolek, & Luttbeg, 2014).

Native to North America, invasive populations of *P. acuta* have been introduced around the world and are now established on six continents, typically in rich, disturbed, and lentic environments (Albrecht, Kroll, Terrazas, & Wilke, 2009; Dillon, Wethington, Rhett, & Smith, 2002). The snail is simultaneously hermaphroditic and capable of self‐fertilization (Dillon, McCullough, & Earnhardt, 2005), although outcrossing is preferred (Escobar et al., 2011; Wethington & Dillon, 1997). Generation time in the laboratory can be as short as 6 weeks (Wethington & Dillon, 1993), although wild populations typically complete only one or two generations per year, both effectively semelparous (Life cycles A or C of Dillon, 2000: 158).

We originally sampled the population of *P. acuta* inhabiting the Quarterman Park “Duck Pond” in North Charleston, SC, as part of a 1991 population genetic survey of the Carolina Sea Islands (population “NPK” of Dillon & Wethington, 1995). The population demonstrated allozyme variation interpretable as the product of codominant alleles segregating in Mendelian fashion at three loci: isocitrate dehydrogenase (Isdh), 6‐phosphogluconate dehydrogenase (6pgd), and esterase‐3 (Est3). Mendelian inheritance at the (strong, slow) Est3 locus has been confirmed by Dillon and Wethington (1994).

The Duck Pond drains directly into the brackish Cooper River, effectively isolating the population of freshwater snails it contains by both land and sea. The nearest neighboring population of *P. acuta* is probably that inhabiting the upper, freshwater marshes of Filbin Creek, approximately 2 km north overland. Ducks and other waterfowl doubtless visit both habitats, providing some opportunity for genetic exchange, albeit infrequent. The effects of migration on *N*_e_ have been studied by Gilbert and Whitlock (2015).

## 2 MATERIALS AND METHODS

The Quarterman Park Duck Pond (32.87822, −79.98077) was constructed from a marshy embayment of the Cooper River in the early 20th century. For most of its history, it was tidally influenced and slightly brackish, but recent drainage improvements have rendered it entirely fresh, fed by local runoff. Its area at present is approximately 1.0 hectare, and depth is no more than 2 m.

The pond is maintained by city personnel at irregular intervals and has been kept free of macrophytic vegetation in recent years. Water temperatures can rise above 35°C during summer months, depressing dissolved oxygen to low levels, despite city efforts at artificial aeration. Its population of *P. acuta* reaches maximum density on allochthonous leaves and debris floating at the eastern (windward) end of the pond, at the drain.

We visited the pond each spring from 2009 to 2015, beginning in March, examining debris at the eastern end to qualitatively assess snail densities. If the apparent census size was sufficient to yield several hundred snails with reasonable effort, an annual sample was taken. Otherwise, we postponed the sample and returned a few weeks later. Approximately 150–200 *P*. *acuta* were ultimately sampled every spring, with one exception. The exception was 2012, when the snail population never reached an abundance at which it could be sampled, from March to August.

Snails collected at each sampling year were returned to the laboratory and frozen individually in 80–160 μl of tris tissue buffer for analysis of allozyme polymorphism. We used horizontal starch gel electrophoresis in a TEB8 buffer system to resolve variation at the Est3, Isdh, and 6pgd loci and an aminopropylmorpholine pH 6 buffer system for a second examination of Isdh and 6pgd. Details regarding our electrophoretic methods, including a description of our equipment and recipes for all stains and buffers employed, have been published by Dillon (1992) and Dillon and Wethington (1995).

Allele frequencies and tests of fit to Hardy–Weinberg expectation were calculated using GenePop version 4.5.1 (Raymond & Rousset, 1995; Rousset, 2008). Values of *F*_IS_ were computed using the method of Weir and Cockerham (1984), and exact *p*‐values were by the Markov chain method.

NeEstimator v2.01 is freely available software designed to estimate effective population size using three single‐sample methods and three‐two‐sample (moment‐based temporal) methods (Do et al., 2014). Among the single‐sample methods, Gilbert and Whitlock (2015) reported that the linkage disequilibrium (LDNe) method of Waples and Do (2008) consistently returned the lowest root square mean error across the range of effective population numbers simulated, absent migration.

The three‐two‐sample methods implemented by NeEstimator 2.01 employ the standard temporal method (ST) of Waples (1989), with different approaches to computing standardized allele frequency variance: the Fc of Nei and Tajima (1981), the Fk of Pollak (1983), and the Fs of Jorde and Ryman (2007). The simulations of Gilbert and Whitlock (2015) suggested that all three of these two‐sample methods perform with equivalent efficiency. Thus, we elected to estimate the effective population size of the Quarterman Park *P. acuta* population using four approaches: LDNe, STFc, STFk, and STFs. Jackknife methods were used to calculate 95% confidence intervals (CI) for all *N*_e_ estimates.

## 3 RESULTS

**Figure 2:**
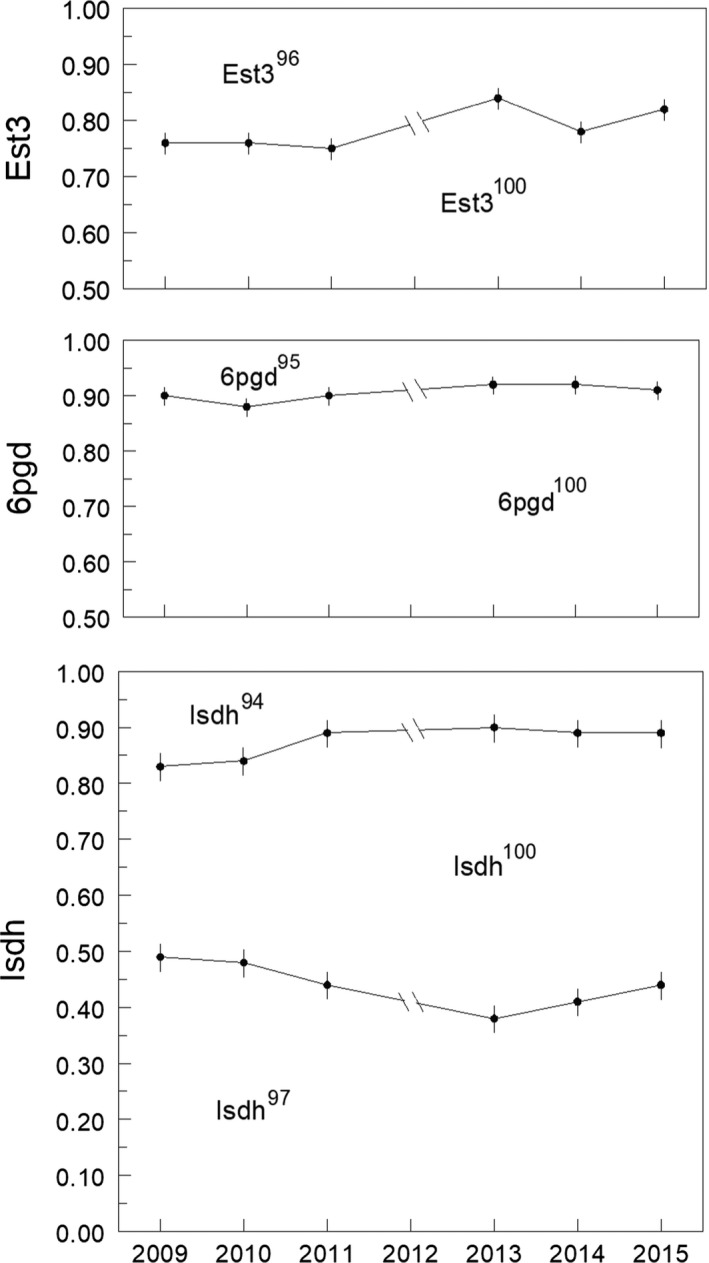
Allele frequencies at three allozyme‐encoding loci in the Quarterman Park *Physa acuta* population, sampled over 7 years. Bars are 95% CI

We resolved allozyme variation apparently encoded by two codominant alleles at the Est3 and 6pgd loci and three codominant alleles at Isdh. These alleles were named by the mobility of their allozyme bands relative to the standards set by Dillon and Wethington (1995) and plotted by their frequencies in Figure 2.

Sample sizes, values of *F*_IS_, and values of *p* from goodness‐of‐fit tests to Hardy–Weinberg expectation are reported in Table 1. Over the entire data set of 3 loci × 6 years, 15 of the values of *F*_IS_ were positive, some strikingly so, and three were slightly negative. Four values of *F*_IS_ suggested significant heterozygote deficiencies (three at the Est3 locus and one at 6pgd), although not significant after Bonferroni correction.

**Table 1:** Sample sizes, values of *F*_IS_, and values of *p* from goodness‐of‐fit tests to Hardy–Weinberg expectation at three allozyme‐encoding loci analyzed for the Quarterman Park *Physa acuta* population 2009–2015. The bottom two rows report single‐sample estimates of effective population size. Inf., effectively infinite

	2009	2010	2011	2013	2014	2015
Est3						
*N*	217	217	217	184	217	186
*F*_IS_	0.054	0.149	−0.002	0.184	0.207	0.060
*p*	.461	.034	1.00	.022	.005	.465
6pgd						
*N*	217	217	216	186	217	186
*F*_IS_	−0.009	0.157	−0.057	0.053	−0.023	0.088
*p*	1.00	.029	.700	.366	1.00	.204
Isdh						
*N*	210	215	215	186	217	186
*F*_IS_	0.032	0.069	0.114	0.037	0.090	0.107
*p*	.631	.568	.138	.673	.086	.079
LDNe	Inf.	Inf.	125.8	44.0	190.8	9,752
95% CI			34.6	10.8	2.0	51.0

The six single‐sample estimates of effective population size based on linkage disequilibrium between the three loci are reported at the bottom of Table 1, with 95% CI. Table 2 shows the five‐two‐sample estimates of effective population size based on allele frequency variance across pairs of consecutive samples. Each two‐sample calculation was performed using Fc, Fk, and Fs methods, yielding 3 × 5 = 15 *N*_e_ estimates, with confidence intervals.

**Table 2:** Two‐sample estimates of effective population size calculated for the Quarterman Park *Physa acuta* population 2009–2015, using three different approaches to estimate standardized allele frequency variance. Inf., effectively infinite

	2009–2010	2010–2011	2011–2013	2013–2014	2014–2015
STFc	Inf.	131.5	49.8	255.8	Inf.
95% CI		9.2	8.1	7.3	
STFs	Inf.	113.6	40.6	205.4	15,361
95% CI		34.8	17.3	35.9	453
STFk	Inf.	104.7	56.5	354.2	Inf.
95% CI		8.0	9.0	8.1	

## 4 DISCUSSION

Tables 1 and 2 suggest that the effective size of the *P. acuta* population inhabiting the Quarterman Park Duck Pond was strikingly volatile, dipping from infinite in 2009 down to (remarkably consistent) values of 44.0 ± 10.8 by static estimate, or 49.8 ± 8.1 by two‐sample estimate, and then back up to infinity again. The dramatic fluctuation in *N*_e_ seemed to correspond to a fluctuation in apparent census size noticeable in the field, from (surely) thousands in the spring of 2009 to a very few in 2012, and then back up to thousands.

The spring of 2012 was exceptionally warm in North Charleston. The average temperature recorded by the National Weather Service over the month of March, 2012, was 65.3°F (18.5°C), the second highest mean March temperature in the 80‐year record. It was our pond side observation that the *P. acuta* population at Quarterman Park never bloomed in the spring of 2012, which seemed to depress its size for the remainder of the year.

Both the effective size and the apparent census size of the *P. acuta* population in the Quarterman Duck Pond apparently returned to many thousands (at minimum) in just 2 years, perhaps four generations. So the most obvious hypothesis to account for the depression in effective size observed between 2011 and 2013 would be a population bottleneck. But *N*_e_ is not expected to recover from such a striking bottleneck event until many generations have passed, absent migration (Caballero, 1994).

A less obvious hypothesis might be fluctuation in the selfing rate, such that the population of *P. acuta* in Quarterman Park shifted from outbreeding to inbreeding and then back again over the study interval. The only estimates of *N*_e_ remotely comparable to ours in the published literature are the works of Meunier et al. (2004) and Trouve et al. (2005) on European populations of the preferentially selfing *Lymnaea (Galba) truncatula*. The French populations studied by the former authors generally demonstrated *N*_e_ < 30, and the Swiss populations studied by the latter *N*_e_ < 10. But the selfing rates inferred for all *L. truncatula* populations in both studies, estimated from *F*_IS_, consistently exceeded 80%. The heterozygosities we observed in our study population of *P. acuta* did not vary from expectation through our 7‐year observation period.

We suggest that cryptic population subdivision may be the most likely hypothesis to account for the apparent volatility of *N*_e_ in our 7‐year record. We sampled the Quarterman Park population of *P. acuta* at the east end of the pond for convenience. Snails were also observed elsewhere around the entire margin of the one‐hectare pond, but not in densities sufficient to sample in the quantities required. Perhaps the striking dip in apparent population census size we observed in 2012 was localized on the east end, and its subsequent recovery was due to immigration from elsewhere within a subdivided population.

Some of the most influential studies of population subdivision published to date have been conducted using land snail models. Cain and Currey (1963) described small‐scale variation in the frequencies of shell color morphs in the English land snail *Cepaea* as “area effects,” attributing the phenomenon to genetic drift. Among the earliest examples of parapatric speciation to be proposed was that of Murray and Clarke (1968), working with the localized clines in shell color polymorphisms demonstrated by the tropical land snail, *Partula*. Selander and Kaufman (1975) documented significant variance in the frequencies of allozyme‐encoding genes in a population of the land snail *Helix (Cornu) aspersa* inhabiting two city blocks in Bryan, Texas. Some of this variance could be correlated with observable barriers to dispersal such as roads or walkways, but some could not.

The published literature also contains many reports of significant values of Fst among subpopulations of freshwater snails. Most of these studies have been conducted where subpopulations are divided by identifiable barriers to dispersal, however, which is not the case in the Duck Pond at Quarterman Park. Jarne and Delay (1990) estimated values of *F*_IS_ and *F*_ST_ within and among several subpopulations of *Lymnaea peregra* (“*Radix balthica*”) sampled from Lake Geneva, reporting large values of the former but rather small values of the latter. Dybdahl and Lively (1996) reported significant values of *F*_ST_ between subpopulations of *Potamopyrgus antipodarium* sampled within several New Zealand lakes.

Like *Lymnaea truncatula*, the freshwater pulmonate snail *Bulinus truncatus* is a preferential self‐fertilizer, Viard, Justy, and Jarne (1997) estimating selfing rates across 38 West African populations from 80% to 100%. The authors noted surprising variation in the levels of microsatellite polymorphism demonstrated by these populations, however, some showing no polymorphism at any of the four loci examined, others averaging over 10 alleles per locus. Viard and colleagues suggested some unseen variation in population sizes as a possible explanation for this phenomenon.

Puurtinen, Knott, Suonpaa, Van Ooik, and Kaitala (2004) reported significant positive correlations between the microsatellite polymorphism demonstrated by eight Finnish populations of the preferentially outcrossing freshwater pulmonate, *Lymnaea stagnalis*, and several measures of fitness, including maturation age and fecundity. The authors suggested that their measures of genetic variation might indirectly estimate effective population size, with lower values of *N*_e_ promoting the random fixation of deleterious alleles. But Puurtinen and colleagues could not demonstrate a correlation between either genetic variability or population fitness and the current densities of the snail populations they sampled.

It should be cautioned that the number of genetic markers employed for the present study was small. The effectively infinite population sizes we estimated 2009–2010 and 2015 might result from fluctuating sampling variance. But our results, suggesting as they do striking volatility in the effective population size of a common and widespread pulmonate snail, offer a potential resolution to quandaries such as those reported by Viard, Puurtinen, and their colleagues.

## CONFLICT OF INTEREST

None declared.
